# Draft Genome Sequence of a *Chitinimonas* Species from Hudson Valley Waterways That Expresses Violacein Pigment

**DOI:** 10.1128/MRA.00683-19

**Published:** 2019-08-29

**Authors:** Brooke A. Jude

**Affiliations:** a Reem-Kayden Center for Science and Computation, Program of Biology, Bard College, Annandale-on-Hudson, New York, USA; University of Maryland School of Medicine

## Abstract

*Chitinimonas* spp. are Gram-negative bacilli that are observed in freshwater and soil sources. A number of *Chitinimonas* species have been characterized, including the green-pigmented Chitinimonas viridis. The isolate described here, BJB300, was obtained from a freshwater source in the Hudson Valley, NY. BJB300 is the first *Chitinimonas* isolate expressing violacein, a pigment with biotherapeutic potential.

## ANNOUNCEMENT

*Chitinimonas* bacteria are Gram-negative, motile bacilli that are found in freshwater and soil and include Chitinimonas koreensis, Chitinimonas taiwanensis, and Chitinimonas viridis (1–3). Isolates are often associated with chitin or organisms with chitinous carapaces (2). *Chitinimonas* spp. are capable of producing chitinases, whose production in other organisms is regulated by quorum sensing (4).

Hudson Valley (NY) freshwater samples were cultured at 22 to 25°C on Reasoner’s 2A (R2A) medium, and they displayed vibrantly colored bacterial isolates, including violet colonies (5). The pigment violacein is produced via the expression of a five-gene biosynthetic operon, *vioABCDE* (6). Since its initial characterization, violacein has been studied for its utility in biotherapeutics, most notably for its killing effect on invasive chytrid isolates (7).

*Chitinimonas* sp. strain BJB300 was isolated from a freshwater source on R2A agar and incubated at 22 to 25°C for 48 hours. The isolate grew as diffuse, irregularly shaped, violet-pigmented colonies that could be maintained successfully on R2A agar and 1% tryptone yet is unable to grow on Lennox lysogeny broth (8).

Genomic DNA extraction was completed with a Puregene yeast/bacteria kit (Qiagen). A 150-bp paired-end Illumina library was generated and sequenced on an Illumina HiSeq 4000 sequencer (Wright Labs, Huntington, PA), resulting in 2 Gbp of sequence. DNA was also isolated using the DNeasy blood and tissue kit (Qiagen), and a library was constructed (see SRA accession numbers), without shearing, using the Nanopore rapid sequencing kit (catalog number SQN-RAD004; Oxford). The library was sequenced with the Nanopore MinION device (Oxford), yielding 18.5 million bases. Reads from both sequencing runs were archived through the NCBI and uploaded to the Galaxy Web platform, using the public server at http://usegalaxy.eu, for analysis (Table 1). All programs were run on Galaxy-EU using standard installations except where noted. Illumina sequences were analyzed with FastQC (9) and trimmed using fastp (10), while Nanopore adapters were trimmed using Porechop (11). Unicycler (v. 0.4.6) was used for assembly, removing contigs shorter than 500 bp in length (12, 13). Sequences were mapped back to the assembly using Bowtie 2 and visualized with Tablet, with all contigs having least 230× coverage (14, 15).

**TABLE 1:** Accession numbers for sequence and assembly files

Category	Accession no.	Description
Umbrella project (BioProject)	PRJNA326269	Violacein-producing microbial strains
BioProject	PRJNA263247	*Chitinimonas* sp. strain BJB300 genome sequencing
BioSample	SAMN11883734	Microbe sample from *Chitinimonas* sp. BJB300
SRA-Nanopore	SRX6461400	SRR9151613
BioSample	SAMN03098007	Microbe sample from *Chitinimonas* sp.
SRA-Illumina	SRX3373346	SRR6267149
Unicycler assembly (GenBank)	VDCU00000000	

The draft genome is 111 contigs. The *N*_50_ value of the assembly is 246,315 bp. The genome size is predicted to be 5.04 Mbp, with a G+C content of 54.56%. The G+C percentages in the literature for *Chitinimonas* species range from that for *C. viridis* (59.8% G+C) to that for *C. koreensis* (65.0% G+C) (1, 3). Analysis with PlasFlow identified 33 potential plasmid sequences, many with G+C contents divergent from the reported average (16).

Contigs were annotated using Prokka (v. 1.13.3) (17), RAST*tk* (https://www.patricbrc.org) (18, 19), and the NCBI Prokaryotic Genome Annotation Pipeline (20) Annotations averaged 4,652 coding sequences (CDS). A BLAST search of the 16S rRNA found that it was 94% identical to that of *C. koreensis.* As expected, the violacein operon was identified, describing the purple colony pigmentation. Additionally, a chitinase gene was noted, pointing to a functional ability to degrade chitin similar to that of other *Chitinimonas* isolates.

This report places *Chitinimonas* into a group of strains capable of producing violacein, enlarging the cohort of bacterial strains available for bioremediation and biotherapeutic purposes. Further analysis of this strain and its biological properties is ongoing.

### Data availability.

SRA files for Illumina sequencing (SRA accession number SRS2670610) have been deposited, as well as those for Nanopore sequencing (SRA accession number SRX6461400). This whole-genome shotgun project has been deposited at DDBJ/ENA/GenBank under the accession number VDCU00000000. The version described in this paper is the second version, VDCU02000000.
